# Pathology Founded on the Natural System of Anatomy and Physiology; a Philosophical Sketch, &c.

**Published:** 1842-01

**Authors:** 


### Art. XIII.

*Pathology founded on the Natural System of Anatomy and Physiology; a Philosophical Sketch, &c.* By Alexander Walker. Second Edition.—*London*, 1841. 8vo, pp. 162.

Mr. Walker is doubtless an ingenious and subtle thinker, and if he had any purely abstract subject to deal with, he might prove a very seductive reasoner, and one by no means easy to confute. In such abstract subjects, the chief end of argument is to apply generally-acknowledged principles to the solution of particular questions. In the physical sciences, on the contrary, we have yet arrived at few general principles, and the great object is to enlarge their number by the accumulation and comparison of facts. Now it unfortunately happens that the great difficulty of ascertaining facts in physical science renders their accumulation a very tardy process; and hence men of a lively genius are apt to educe what they call general principles from very partial induction, and when they come to apply them to the solution of a question, they are apt to overlook all facts opposed to principles which they think universal, because they have an all-pervading influence on their own minds. We fear that Mr. Walker belongs to this class of philosophers; for it appears to us that there are very few positions in the argumentative and really clever production before us, that might not be upset by a reference to the most familiar facts. There is one feature in this work of which we entirely approve, namely, that the author, after the manner of many excellent French writers, has given a recapitulation of the conclusions at which he has arrived. This proceeding is to be commended, *first*, because it shows that the author understands his own meaning, which is not the case with all authors; and *secondly*, because it shows that he is not afraid to subject his disrobed opinions to the criticism of the world, thereby affording a strong presumption that he himself believes what he says, which also is far from being universal among authors. As reviewers, moreover, we have reason to applaud this proceeding, because it saves us a great deal of trouble: we beg, however, to assure Mr. Walker that in availing ourselves of his "summary" we have by no means neglected the task of reading, with minute attention, every syllable of his work, the chief contents of which we will now proceed to analyse.

1. Anatomy is the basis of physiology, and physiology the basis of pathology: this being admitted, a classification which places the objects of anatomy in a true and natural relation to one another, will apply in like manner to the objects of physiology and pathology.

According to Mr. Walker's method, anatomy is distinguished into three parts: that which regards the locomotive organs; that which regards the vital or nutritive organs; and that which regards the mental or thinking organs. In order to classify the objects of animal physiology, it is only necessary to substitute the term "functions" for that of "organs," and this department of science resolves itself into the consideration of the locomotive, nutritive, and mental functions. In pathology, again, we have merely to substitute "diseased functions" for "healthy functions," and we have three classes, embracing diseases of the locomotive functions, of the vitalor nutritive functions, and of the mental or thinking functions.

In the abstract, we see no particular objection to this arrangement; yet, we imagine, every one but Mr. Walker will at once perceive that it is entirely inapplicable to the present state of knowledge, and that its adoption would immediately bring medicine to a dead stand; since it would imply that our science must be utterly irrational, and unavailable, unless when founded on a perfect knowledge of the structure and functions of the animal body, to which physicians have certainly not yet attained, and to which we think it probable they never will attain. We admit freely that our aim should always be to connect medicine as closely as possible with anatomy and physiology, but if we were to reject every pathological view, and every therapeutical indication, which could not be made to square with our author's arrangement, or any other of the like nature, we apprehend that nineteen twentieths of our present supposed knowledge would be brought to nought. Moreover it seems to us not a little extraordinary that Mr. Walker should insist so rigidly on such an arrangement, because, as we shall presently find, his *therapeutics* are based on two very broad principles, one of which, namely, *similia similibus curentur*, has no appreciable relation to anatomy or physiology, or any pathological doctrine derived from them, but, (in as far as its truth extends) rests upon experience alone.

It would have been well if our author had given us a few practical examples of pathological views founded immediately on the functions, and thence on the structure of parts; though we must confess that if all his notions on physiology be as hypothetical as those contained in the following extract, we think medicine had better be allowed to remain on its present avowedly and imperfect heterogeneous basis.

"Under the vital or nutritive organs, I class, first, the lacteals, fine tubular vessels which absorb nutritious matter from the food taken into the intestines, and carry it towards the heart, to be converted into blood; second, the blood-vessels, which circulate the blood thus formed; and third, the glands and other parts which secrete or deposit not only the substances composing the organs, but the various animal products; all of these organs consisting of *tubes* which exercise only a minute peristaltic or *pulsating motion*, and of which the trunk of the body is the centre and principal seat. Under the mental or thinking organs, I class, first, the organs of sense, the eye, ear, &c., which receive impressions from external bodies; second, the brain, which perceives, compares, reflects, &c.; and, third, the cerebel or little brain situated below the back part of the greater brain and above the neck, which wills and consequently throws the muscles into those actions which fulfil its purposes; all these organs consisting of *tubules* and *fibres* in which *no motion* is *visible*, and which occupy chiefly the head." (p. 13.)

We should be glad to know where Mr. Walker learned that the tubes which constitute a gland exercise a *peristaltic or pulsating* motion? Did he ever see the tubes at work? The use of the word "or," and the italics in the above extract, would seem to imply that it is immaterial whether the motion be of the peristaltic or of the pulsatory kind. Now, with respect to the motion best adapted to the function of the glands, we must profess our entire ignorance; but of the general convertibility of these two kinds of motion for the performance of vital functions, we have such serious misgiving that we should be not a little alarmed at the prospect of our intestinal tube assuming a *pulsatory*, and our heart a *peristaltic* action. Again, though Mr. Walker's researches may have satisfied him that the cerebellum *wills*, we imagine that physiologists in general will not be disposed to admit his *opinion*, for such merely it is, as affording the solution of a question on which so much experimental research has been spent in vain. Reil and Rolando say, that the cerebellum is a galvanic pile, or something very similar, and that it generates the nervous energy; Flourens says, it is the combiner and regulator of the voluntary movements; Gall says, it is the organ of sexual desire; Mr. Walker says it is the organ of the will; and WE protest that we have not the remotest idea what its function may be. Is it on such conjectural grounds as these that Mr. Walker would found his "natural system of medicine?" We can assure him that it is difficult enough to bring the actual truths of anatomy and physiology into the relations with medicine, which are so much to be desired; but if the subject is to be mystified by mere conjectures in these sciences, medicine must soon become as "the baseless fabric of a vision."

2. In every disease there are two kinds of symptoms, *morbid* and *curative;* the former being the signs of the disease, and the latter of the reaction which nature establishes in order to get rid of the disease: these two kinds of symptoms are therefore directly opposed to each other. Mr. Walker conceives that the contentions of the *Heteropathists* and *Homœopathists,* and the errors into which both have fallen, are attributable to the ignorance which has hitherto prevailed of the foregoing distinction of symptoms into *morbid* and *curative.* He believes that he is the first writer who has clearly stated this distinction, as universally applicable to the phenomena of disease. We grant that he may have stated it in a more emphatic and aphoristic manner than has been done before; but we maintain that the distinction did nevertheless virtually pervade the pathology and practice of the Greek school, and has been tacitly recognized by almost all their successors.

We presume Mr. Walker is too philosophical a man to stickle about terms, and we can assure him that his "curative symptoms" were very clearly indicated by Galen under the name of *εργα—operations.* We select a passage near the end of the book *περι αιτιων συμπτωματων,* which will, we hope, sufficiently justify our assertion. We translate it as literally as possible from the Greek text;

"But particular attention should here be given to discriminate accurately between *symptoms,* and the *operations of the living frame;* for they often resemble each other so closely, that a *symptom* may be mistaken for an *operation,* and an *operation* for a *symptom.* And unless any one be of a sound judgment in this matter, he will misrepresent our arguments through his own mistakes. For, if he propose as a standard, the quantity, or quality, or kind, of the natural excretions (so those are called which occur in healthy persons), and, contemplating these, judge thence with respect to symptoms, he will be oftentimes deceived; since a sweat, or alvine discharge, or flow of urine, much more profuse than natural, sometimes occurs in sick persons, not only without lesion of any action, but through the power and providence of the living frame; for some things, even though they may be altogether preternatural, as flow of blood from the nostrils, or vomiting, or purging of blood, or hemorrhois, or anything else of the kind, are yet not contrary to nature if they occur seasonably, and *they are evidently seasonable when that which aggrieves the system is expelled.* Keeping therefore to what we stated at the commencement, that symptoms are lesions of actions, *none of those things which aid the system belong to this kind of symptoms, for each of them seems rather an operation of nature than any sort of lesion.*" (GALEN. ed. Basil. tom. iii. p. 218.)

It may be said that this notion of Galen's related only to critical evacuations, and therefore cannot be considered as of general application; but it is to be remembered that he followed Hippocrates, in referring all diseases to excess, diminution, depravation, or wrong direction of the humours, and hence that a discharge of the offending humour entered very largely into his ideas of curative processes whether natural or artificial.

3. According to Mr. Walker there is a very simple criterion for the *discrimination* of *morbid* and *curative* symptoms; namely, the presence or absence of *pain.*

"Pain," he says, "is an act of the nervous system, the outcry of life when suffering under morbid symptoms, and the portion of the vis medicatrix naturæ exciting to the production of curative symptoms. Pain is therefore *the means of distinguishing morbid from curative symptoms.* Gentle reaction, slight injection, or incipient inflammation, is the consequence of pain, the reaction of the vital system, the instrument of the *vis medicatrix naturæ, in the cure of the disease.* Medicines being perfectly simple, and their power being determined by their effects on persons in health, as well as in disease, their administration will follow as far as possible the order of phenomena. Where the cause and morbid symptoms can at once be removed, the law *contraria* will be acted upon, as in taking a thorn from the flesh, adding an alkaline antidote to a metallic poison, &c., and the law *similia* may not be acted upon at all. Where the morbid and curative symptoms follow in successive stages, the morbid symptoms will first be attacked by the law contraria, and then the curative will be guided by the law similia, as in the cold and the hot fit of fever. Where the same medicament at once attacks the morbid, and aids the curative symptoms, both laws will at once be acted upon, as by at once removing the irritating matter and aiding the ejection in diarrhœa. Thus the law contraria may be acted upon singly, or both laws in succession, or both laws at the same time and by a single medicament. The *doses* of medicines ought to be regulated by their intended application to the morbid or to the curative symptoms respectively." (pp. 157-8.)

If all this were correct, it would amount simply to the most important discovery ever made in practical medicine; but five minutes' attention to the most familiar phenomena of disease will suffice to show the insufficiency of Mr. Walker's criterion. For example, when the painful symptoms of inflammation of the brain subside into the state of profound coma, there is a cessation of suffering; but would any practitioner thence infer a curative effort of nature? or would he not, on the contrary, infer the probability of a speedily fatal termination? Again, in a case of puerperal peritonitis, would the cessation of pain, accompanied with increased tumefaction of the abdomen and sinking of the pulse, lead the practitioner to hope that nature was making a curative effort, or would it not rather indicate the approaching dissolution of the patient? A hundred other instances of the same kind present themselves as we pass in review any catalogue of diseases. Take the example of a boil. Here an exceedingly painful kind of inflammation is set up by the *vis medicatrix* to get rid of the dead core, and this is succeeded by suppuration, which increases the torment sevenfold till the skin gives way: immediate relief then follows. Now we submit that these painful actions are nevertheless curative ones. Lastly, when the vitality of the ovum is destroyed at an early period of gestation, are the painful contractions of the uterus, by which it is expelled, *morbid* or *curative?*—surely the latter. The presence of pain, then, is no proof that actions may not be of a curative kind, or that they are what our author calls *morbid.* It would be wasting our own time and that of our practical readers to multiply such examples. All we can say of Mr. Walker's criterion is, that we most heartily wish it were true, but are *painfully* convinced by facts that it is otherwise.

From his reasoning on the nature of symptoms, our author concludes that the laws "*contraria contrariis*" and "*similia similibus*" are each applicable to the treatment of disease; that *homœopathy* and *heteropathy* have both a foundation in truth, but that the kind of cases to which they are respectively applicable have not been duly discriminated, and can be so only through the distinction of morbid and curative symptoms.

After giving a much fuller consideration than they are worth to the whims and contradictions of the homœopathists, some of whom he refers to the *Organon* of their grand master for better information concerning the principles they profess, Mr. Walker proceeds to deliver what he thinks the true explanation of the manner in which the so-called homœopathic doses really operate in the cure of disease:

"It may be said that these hypotheses [such namely, as attempt to explain the operation of the homœopathic remedies] are of little consequence. If, however, they had not been felt to be otherwise, they never would have been formed or adopted. The object of such hypotheses is the highly important one of rationally explaining the basis of the law 'Similia similibus curantur'—of showing that it is in harmony with natural phenomena and philosophical reasoning. Unexplained by some hypothesis, the rule itself becomes a mere vague hypothesis, instead of a rational theory; and its advocates assuredly desire to raise it above that condition. I think, indeed, that the true explanation of the action of this law can be given, retaining all its experimental and practical worth, its essential part, and reasoning justly respecting it, while we rectify its language. The reasoning, moreover, which is now to be supplied, is amongst the very simplest and most indisputable in physiology. It has indeed been implied in many of the preceding arguments. As then, the common practice is founded chiefly on a consideration of the morbid symptoms and the maxim 'contraria contrariis curantur;' so is homœopathy founded on a consideration of the curative symptoms and the maxim 'similia similibus curantur.' Its medicines may be said indirectly to act *contrarily* to the *morbid symptoms* —the derangement or disease; but their direct action is *similar* to that of the *curative symptoms,* which they therefore assist. In these efforts of nature, as already said, it is the vital system which becomes the healing one, as in fever, inflammation, &c., and it is the nervous system of life which indicates locality and calls up reaction, by pain and various disturbance. Homœopathic medicines, therefore, neither produce such reaction, nor any such disease as the above-mentioned hypotheses propose; they merely assist the already existing efforts of nature, the curative symptoms; and thus all complex explanation is at once got rid of. Even minute doses thus require no 'increased sensibility for the action of specific irritants;' their action becomes one of the most simple and natural events—it is in harmony with and comes in aid of the efforts of nature, the curative symptoms—it adds their force to its own—it gives them a power paramount to the morbid symptoms or to disease properly so called."(p.97.)

This is doubtless a most ingenious attempt at explanation, but we wonder much that so acute a man as Mr. Walker should have given himself so much trouble to account for the efficacy of the homœopathic administration of medicines, without previously ascertaining *whether it had any efficacy?* We affirm that it has—at least in the vast majority of cases—none, and that the apparent success of homœopathic practice admits of a simple and satisfactory solution on very different grounds from those assumed by our author.

We fully agree with him that homœopathic practice is followed by very desirable results in a variety of cases, but we entirely deny that such results are attributable to the medicines employed. The cases we conceive would have come to the same fortunate issue, though not a particle of medicine of any kind had been administered. To give a man the millionth of a grain of any known substance, however active, is, to all practical purposes, to give him nothing; and we beg to assure Mr. Walker that there are many cases in which every good practitioner knows that his duty is merely to regulate the diet and habits of the patient, and remain, otherwise, a passive spectator of the cure which nature is accomplishing. In such cases the homœopathic practice, which, as far as medicines are concerned, amounts to no practice at all, will be found to answer remarkably well. In truth, *homœopathy*, viewed through the medium of common sense, becomes synonymous with *la médecine expectante*. We have elsewhere expressed our opinion that this kind of medicine finds too little favour in our own country, and we have no doubt that many a patient is now groaning under the energetic ministrations of an English doctor, who would thrive much better on millionths of grains; in other words, if he were let alone.

We have now given the fullest consideration which our limits would allow, to the opinions expressed in Mr. Walker's book. It contains many original remarks on various subjects, from which we have derived some edification and much amusement. In common with most theorists, the author attaches too much importance to the theories of others, and assails with mis-spent vigour positions fit only to be laughed at. Thus he quotes Dr. Dickson, of *fallacious* celebrity, just as if he were an authority, and enters into an elaborate confutation of Hahnemann's spiritualization of disease. He winds up his remarks on the latter subject with a passage in which his vivacity seems to have carried him slightly beyond the limits of urbanity, but which, withal, is so capital, that we cannot help extracting it:

"In this country, when even the lowest classes, in their apprehension of events, consented to give up the easy explanation afforded by ghosts, science also began to relinquish its ghosts; its electrical, magnetic, and other spirits. But among the Germans, at the present day, ghosts pervade all life, all legends, all poetry, and all philosophy. With their aid, our doctors would soon reach the incantations of magic, and would rival the Peeaïs or priest-doctors of the American Indians. Some indeed of the more spiritually inclined have already followed these their worthy rivals, in the adoption and practice of Mesmerism, with all its contemptible antics and disgusting humbug!"

In conclusion, we can conscientiously recommend Mr. Walker's book to the perusal of all our readers. His mode of reasoning is not indeed adapted to the establishment of scientific truth, but he is continually throwing out ideas which may aid others in its pursuit, so that his work, though full of fallacies, is far from being useless.

---

### Art. XIV.

*Transactions of the Pharmaceutical Society.* Edited by Jacob Bell. Nos. I-VI.—*London,* 1841. 8vo, pp. 293.

